# Oral *Centella asiatica* Extract Attenuates UVB-Induced Skin Photoaging via Antioxidant, Anti-Inflammatory, and Extracellular Matrix-Preserving Effects in Hairless Mice

**DOI:** 10.3390/ijms27010204

**Published:** 2025-12-24

**Authors:** Yean Jung Choi, Eun-Chae Cho, Seungtae Lim, Jaemin Lee, Jaewoo Bae, Tae Kyu Oh, Jae Kyoung Lee, Eun Ji Kim

**Affiliations:** 1 Department of Food and Nutrition, Sahmyook University, Seoul 01795, Republic of Korea; yjchoi@syu.ac.kr (Y.J.C.); ec.cho0201@gmail.com (E.-C.C.); ljm0202@syuin.ac.kr (J.L.); 2 Institute of Nutritional Physiology and Molecular Nutrition, Sahmyook University, Seoul 01795, Republic of Korea; 3 Research Institute, SRE Service Co., Ltd., Chuncheon 24232, Republic of Korea; stlim@sres.co.kr; 4 3H LABS Research Institute, 3H LABS Co., Ltd., Goyang 10391, Republic of Korea; jerry@3h-labs.com (J.B.); owen@3h-labs.com (T.K.O.); ljk1200@3h-labs.com (J.K.L.)

**Keywords:** *Centella asiatica*, oral administration, UVB-induced photoaging, antioxidant enzymes, collagen preservation, anti-inflammatory effect, hairless mice

## Abstract

*Centella asiatica* exhibits antioxidant, anti-inflammatory, and dermal-regenerative activities, yet the in vivo efficacy of an orally administered, dose-standardized extract against ultraviolet B (UVB)-induced photoaging has not been fully elucidated. This study investigated the protective effects of a chemically standardized *C. asiatica* extract (sCAE; 70 mg/g asiaticoside) in UVB-irradiated Skh:HR-1 hairless mice. Animals received oral sCAE (40 or 80 mg/kg/day) for eight weeks during repeated UVB exposure. Comprehensive assessments—including skin biophysical measurements, histological analysis, ELISA, and gene expression profiling—were performed to characterize dose-dependent responses. sCAE significantly reduced wrinkle formation, transepidermal water loss, malondialdehyde accumulation, and pro-inflammatory cytokines, while enhancing skin hydration, elasticity, antioxidant enzyme activities, and collagen expression. It also restored hyaluronic acid, ceramide, and their biosynthetic genes, and suppressed matrix metalloproteinase-1 and -9. Notably, the higher dose (80 mg/kg) consistently shifted key parameters toward normal levels, demonstrating a clear dose–response effect. These findings provide the first integrative in vivo evidence that orally administered, asiaticoside-standardized *C. asiatica* extract mitigates UVB-induced photoaging by concurrently improving barrier lipids, extracellular matrix integrity, inflammation, and oxidative stress, supporting its potential as a nutricosmetic agent for skin health.

## 1. Introduction

The skin functions as the body’s largest protective barrier and is continuously exposed to environmental stressors, including pollutants, lifestyle factors, and ultraviolet (UV) radiation [1,2]. Among these, UVB radiation is a major extrinsic driver of premature skin aging, or photoaging, which manifests as wrinkles, pigmentation, and loss of elasticity due to cumulative oxidative, inflammatory, and extracellular matrix (ECM) damage [2,3,4]. Because photoaging is largely preventable, interest has grown in strategies that strengthen the skin’s endogenous defenses in addition to topical sunscreens. However, concerns regarding the photostability, environmental impact, and user compliance associated with mineral UV filters have stimulated the exploration of systemic, orally delivered photoprotective agents [5,6].

*Centella asiatica*, a medicinal herb widely used in Asian traditional medicine, contains pentacyclic triterpenoids—asiaticoside, madecassoside, asiatic acid, and madecassic acid—known to promote wound repair, collagen synthesis, and antioxidant and anti-inflammatory responses [7,8,9,10,11,12,13,14]. These bioactivities have made *C. asiatica* a prominent ingredient in topical dermatological formulations, where it has demonstrated benefits for skin hydration, barrier restoration, ECM remodeling [8,15,16,17,18,19], and protection against cellular photodamage [20,21,22]. Previous experimental and clinical investigations also indicate that oral *C. asiatica* may exert systemic effects, including improved wound healing and enhanced skin hydration in individuals with diabetes-associated xerosis [23,24].

Despite these advances, important knowledge gaps remain. First, most prior UVB-related studies have focused on topical formulations or on extracts lacking defined phytochemical standardization, making it difficult to compare doses or translate findings to nutraceutical applications. Second, existing oral studies have rarely incorporated a chemically standardized extract with a quantifiable marker such as asiaticoside, which is critical for reproducibility. Third, no previous investigations have simultaneously evaluated multiple physiological domains—including barrier lipids (hyaluronic acid, ceramide), ECM components (collagen, MMPs), oxidative stress markers, inflammatory cytokines, and gene expression—in a single in vivo model under a controlled oral dosing regimen. Finally, dose–response relationships for oral *C. asiatica* in UVB-induced photoaging remain insufficiently defined.

To address these gaps, the present study investigated the effects of an asiaticoside-standardized *C. asiatica* extract (sCAE) administered orally at two doses (40 and 80 mg/kg/day) in UVB-irradiated Skh:HR-1 hairless mice. By integrating biophysical measurements, histology, biochemical assays, and transcriptional profiling, this work provides comprehensive mechanistic insight into the anti-photoaging potential of standardized oral *C. asiatica*. Our findings highlight the capacity of sCAE to improve barrier function, ECM integrity, inflammation, and oxidative stress, thereby supporting its development as a nutricosmetic candidate for systemic photoprotection and skin health.

## 2. Results

### 2.1. Characterization of sCAE Using HPLC

**Figure 1:**
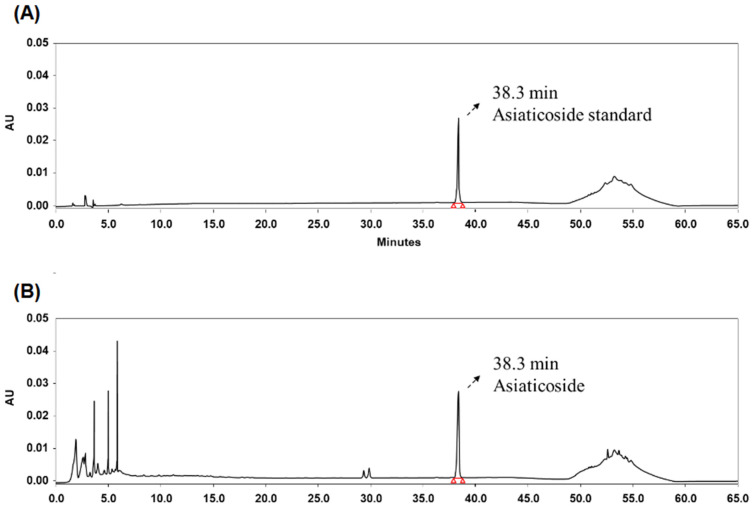
High-performance liquid chromatograms and quantification of asiaticoside in sCAE. High-performance liquid chromatography (HPLC) analysis was conducted. (**A**) Chromatogram of the asiaticoside standard solution (77.42 µg/mL). (**B**) Chromatogram of sCAE (82.67 µg/mL), in which the asiaticoside peak was identified by matching the retention time and UV absorption spectrum with those of the standard. Quantitative analysis indicated that sCAE contained 70 mg/g of asiaticoside.

HPLC analysis was performed to identify and quantify asiaticoside, a major triterpenoid saponin in the sCAE. As presented in Figure 1, both the asiaticoside standard and sCAE exhibited a distinct and well-defined peak at an identical retention time, confirming the presence of asiaticoside in the extract. The analysis was conducted using a photodiode array detector set at 206 nm, and the asiaticoside peak in sCAE was verified by comparison of its retention time and UV absorption spectrum with those of the authentic standard. Quantitative determination, based on a calibration curve constructed using the asiaticoside reference standard, revealed that the sCAE contained 70 mg/g of asiaticoside. These results confirmed that the extract was successfully standardized to asiaticoside as its principal bioactive marker compound, ensuring consistency and analytical reliability for subsequent biological evaluations.

### 2.2. sCAE Mitigates UVB-Induced Photoaging in Skh:HR-1 Hairless Mice

**Figure 2:**
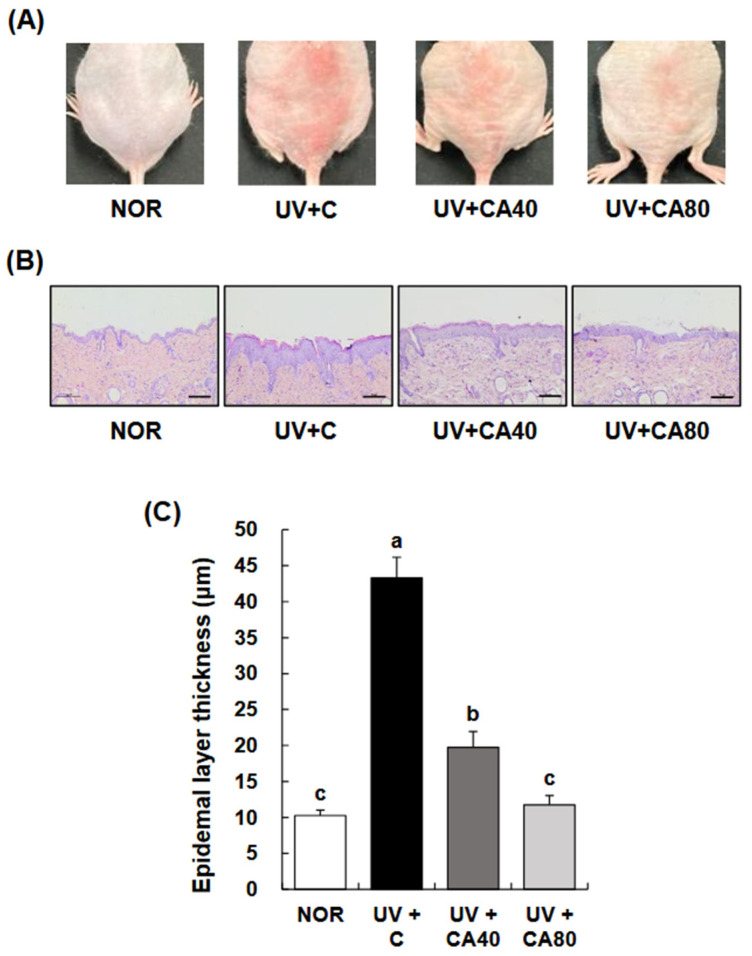
Effect of sCAE administration on photoaging in the dorsal skin in UVB-irradiated hairless mice. All mice, except those in the NOR group, were irradiated with UVB three times weekly for 8 weeks. The mice received sCAE via oral gavage during the same period. (**A**) Representative photographs of dorsal skin were presented (*n* = 10). (**B**) Dorsal skin sections were stained with H&E. Representative H&E-stained images of dorsal skin tissues were presented (n = 10), 200× magnification, Scale bar, 50 μm. (**C**) The thickness of the epidermal layer was measured. Each bar represents the mean ± SEM (*n* = 10). Data are presented as mean ± SEM (*n* = 10 per group). Statistical analysis was performed using one-way ANOVA followed by Duncan’s multiple range test; Tukey’s honestly significant difference (HSD) test was additionally applied for conservative validation. Different letters indicate statistically significant differences at *p* < 0.05.

To evaluate the protective effects of sCAE against UVB-induced photoaging, we assessed macroscopic skin appearance, wrinkle formation, and skin biophysical parameters in Skh:HR-1 hairless mice. As shown in Figure 2A, chronic UVB exposure markedly induced wrinkle formation, rough surface texture, and erythema in the dorsal skin of mice in the UVB-irradiated control (UV+C) group. In contrast, oral administration of sCAE at both 40 mg/kg BW/day (UV+CA40) and 80 mg/kg BW/day (UV+CA80) attenuated these visible signs of photoaging, with the UV+CA80 group showing the most significant improvement.

Histological evaluation of H&E-stained dorsal skin sections (Figure 2B) revealed that UVB exposure induced pronounced epidermal thickening compared to the normal control (NOR) group. In contrast, sCAE administration visibly reduced epidermal hyperplasia in a dose-dependent manner. Quantitative measurement of epidermal thickness (Figure 2C) confirmed these observations, demonstrating that UVB irradiation markedly increased epidermal thickness compared with the normal control (*p* < 0.001). Oral administration of sCAE significantly attenuated UVB-induced epidermal hyperplasia, with both UV+CA40 and UV+CA80 groups showing markedly reduced epidermal thickness compared with the UV-irradiated control (*p* < 0.001 for both). Notably, epidermal thickness in the UV+CA80 group was restored to a level comparable to that of the normal control, indicating near-complete normalization.

**Figure 3:**
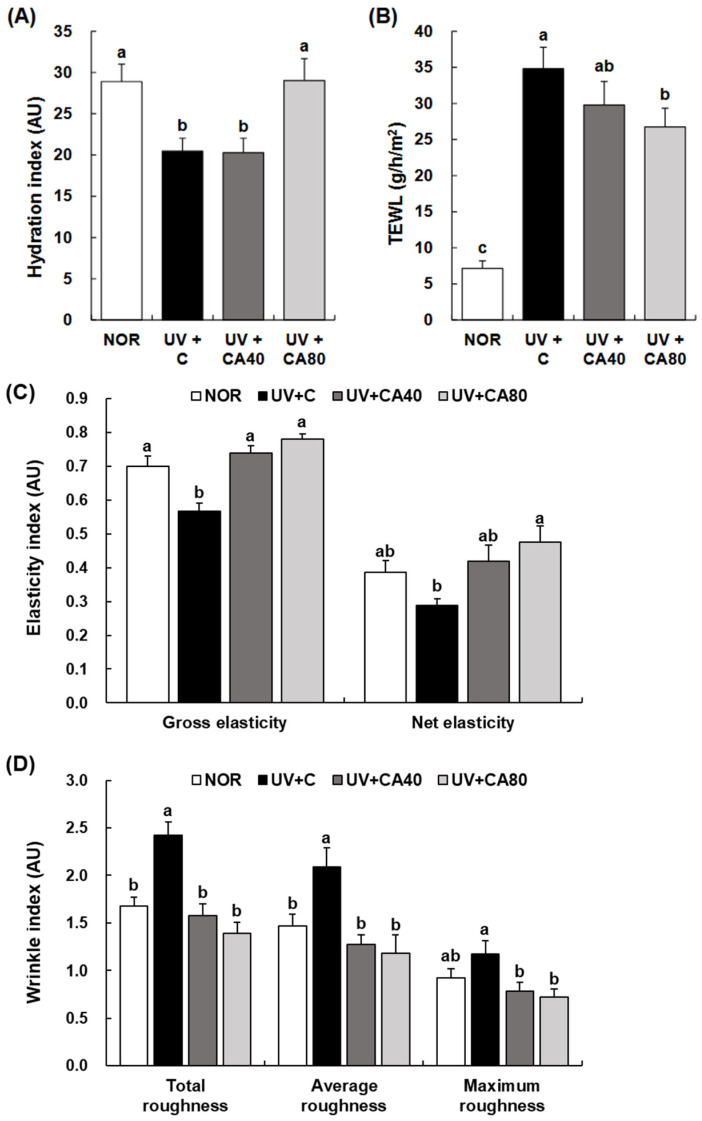
Effect of sCAE on the skin indices reflecting the skin condition in UVB-irradiated hairless mice. All mice, except those in the NOR group, were irradiated with UVB three times weekly for 8 weeks. The mice received sCAE via oral gavage during the same period. Various skin indices were measured using a relevant skin analyzer. (**A**) Hydration index. (**B**) TEWL. (**C**) Elasticity index. (**D**) Wrinkle index. Each bar represents the mean ± SEM (*n* = 10). Data are presented as mean ± SEM (*n* = 10 per group). Statistical analysis was performed using one-way ANOVA followed by Duncan’s multiple range test; Tukey’s honestly significant difference (HSD) test was additionally applied for conservative validation. Different letters indicate statistically significant differences at *p* < 0.05.

Skin biophysical parameters related to barrier function and elasticity were subsequently evaluated (Figure 3A–C). UVB irradiation markedly impaired skin barrier function, as evidenced by increased transepidermal water loss (TEWL) and reduced skin hydration and elasticity. Oral administration of sCAE improved these parameters in a dose-dependent manner. In particular, sCAE at 80 mg/kg significantly enhanced skin hydration (*p* < 0.05) and elasticity compared with the UV-irradiated control, restoring these parameters to levels comparable to those of the normal control. Improvements in gross elasticity were observed at both doses (*p* < 0.001 for both), whereas net elasticity showed a significant increase primarily in the higher-dose group (*p* < 0.01).

Quantitative analysis of dorsal skin replicas further demonstrated that UVB exposure significantly increased wrinkle formation (Figure 3D). Both sCAE-treated groups exhibited significant reductions in total (UV+CA40, *p* < 0.001; UV+CA80, *p* < 0.001) and average (UV+CA40, *p* < 0.01; UV+CA80, *p* < 0.01) wrinkle roughness compared with the UV-irradiated control, while maximum wrinkle depth was significantly reduced in the UV+CA80 group (*p* < 0.05).Notably, across epidermal thickness, skin barrier function, elasticity, and wrinkle parameters, the UV+CA80 group consistently exhibited values closer to those of the normal control than the UV+CA40 group, indicating a clear dose-dependent protective effect of sCAE against UVB-induced skin dysfunction.

For clarity and to facilitate quantitative comparison, the numerical values (mean ± SEM) of all major outcome measures, including skin biophysical parameters, barrier lipids, ECM-related markers, inflammatory cytokines, and oxidative stress indicators, are summarized in Supplementary Table S1.

### 2.3. sCAE Enhances the Expression of Hyaluronic Acid and Ceramide in UVB-Irradiated Hairless Mice

**Figure 4:**
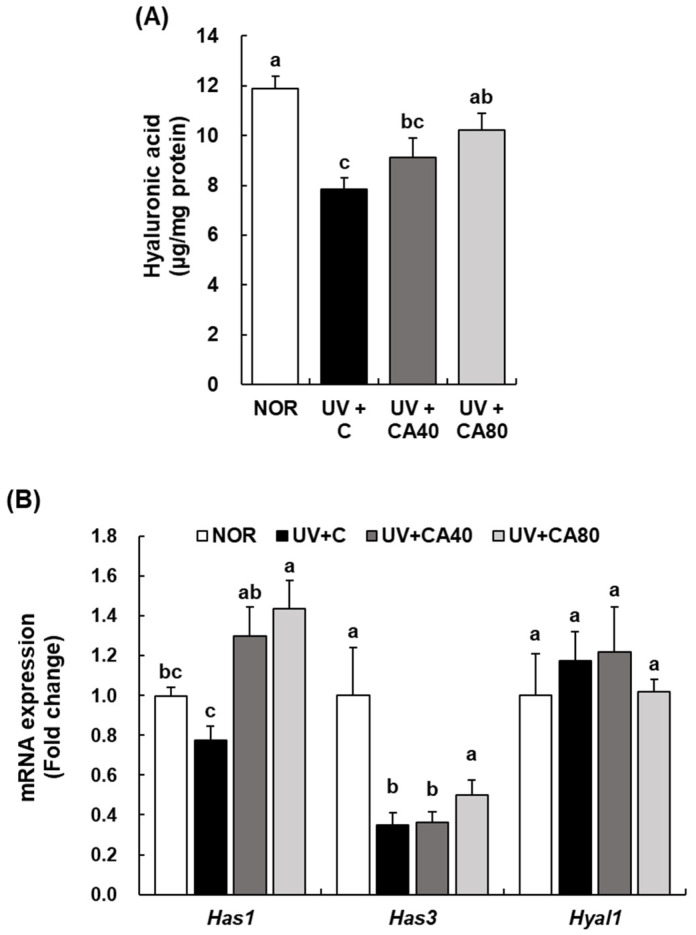

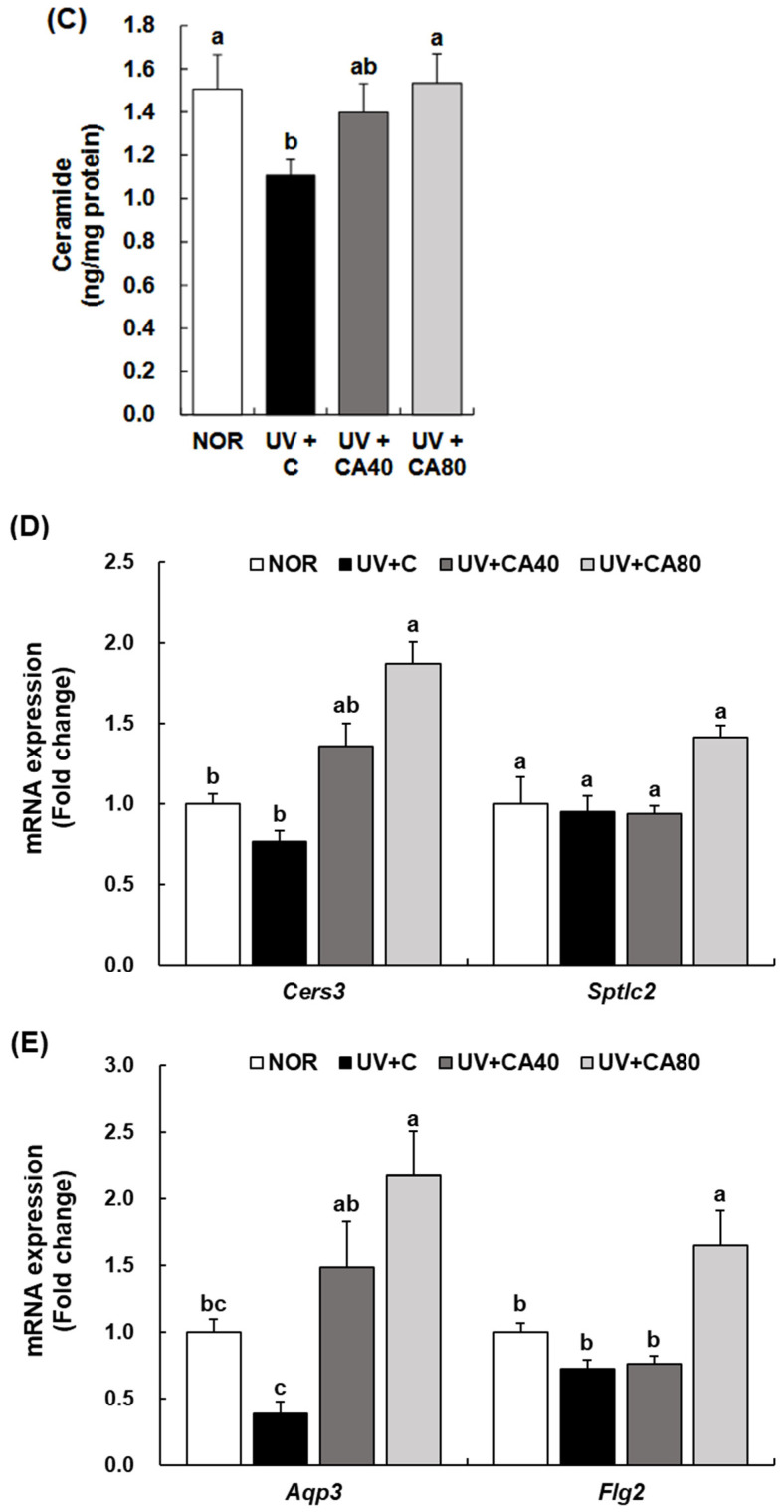
Effect of sCAE on the expression of hyaluronic acid and ceramide in the dorsal skin in UVB-irradiated hairless mice. All mice, except those in the NOR group, were irradiated with UVB three times weekly for 8 weeks. The mice received sCAE via oral gavage during the same period. (**A**,**C**) The dorsal skin was excised and homogenized. The content of hyaluronic acid and ceramide in the skin homogenate was measured using a relevant ELISA kit. (**B**,**D**,**E**) Total RNA in the dorsal skin was extracted, reverse-transcribed, and subjected to real-time PCR. The expression of each mRNA was normalized to that of *Gapdh* and is represented relative to that in the NOR group. Each bar represents the mean ± SEM (*n* = 10). Data are presented as mean ± SEM (*n* = 10 per group). Statistical analysis was performed using one-way ANOVA followed by Duncan’s multiple range test; Tukey’s honestly significant difference (HSD) test was additionally applied for conservative validation. Different letters indicate statistically significant differences at *p* < 0.05.

To assess the effects of sCAE on skin barrier components, we measured the levels of hyaluronic acid and ceramide in dorsal skin tissue of UVB-irradiated hairless mice using ELISA (Figure 4A,C). Hyaluronic acid content was significantly reduced in UVB-irradiated mice compared with the NOR group (*p* < 0.001), indicating UVB-induced impairment of skin hydration. Oral sCAE at 80 mg/kg significantly restored hyaluronic acid levels compared with the UV-irradiated control (*p* < 0.05), whereas the 40 mg/kg dose showed a non-significant trend. Ceramide levels also showed a decreasing trend following UVB exposure; although the overall ANOVA did not reach significance (*p* > 0.05), pairwise analysis indicated a significant increase in the UV+CA80 group compared with UV+C (*p* < 0.05), suggesting substantial biological variability at the protein level. Although both sCAE doses tended to improve barrier-related lipid parameters, the higher dose (80 mg/kg) more effectively restored hyaluronic acid and ceramide levels toward NOR values, supporting a dose–response relationship.

To further explore molecular changes related to skin hydration and barrier regulation, the mRNA expression of related genes, including *Has1* and *Has3* (hyaluronan synthases), *Sptlc2* and *Cers3* (ceramide biosynthesis-related genes), as well as *Aqp3* (aquaporin-3) and *Flg2* (filaggrin-2), was analyzed (Figure 4B,D,E). UVB irradiation significantly downregulated *Has3* expression compared with the NOR group (*p* < 0.01), whereas *Has1* expression was significantly upregulated following sCAE administration, particularly in both doses (*p* < 0.001 and *p* < 0.01, respectively). In contrast, *Hyal1* expression did not differ significantly among groups. For ceramide biosynthesis–related genes, one-way ANOVA revealed a significant group effect for *Cers3* expression. Tukey’s HSD analysis confirmed that the UV+CA80 group exhibited significantly higher *Cers3* mRNA levels compared with the UV-irradiated control (*p* < 0.01), whereas the increase observed in the UV+CA40 group did not reach statistical significance under conservative correction. In contrast, *Sptlc2* expression did not differ significantly among groups by either ANOVA or Tukey’s HSD analysis, indicating a trend-level but not statistically confirmed modulation. Notably, sCAE treatment significantly increased *Aqp3* expression, with the UV+CA80 group showing higher levels than UV+C group (*p* < 0.001). Similarly, *Flg2* expression was significantly elevated in the UV+CA80 group compared with the UV+C group (*p* < 0.01), whereas no significant changes were observed at the lower dose. These findings suggest that although protein-level restoration of hyaluronic acid and ceramide was incomplete, sCAE—particularly at the higher dose—modulated key genes involved in epidermal hydration and barrier function, supporting a transcriptional basis for its barrier-protective effects.

### 2.4. sCAE Alters the Expression of Collagen and MMPs in UVB-Irradiated Hairless Mice

**Figure 5:**
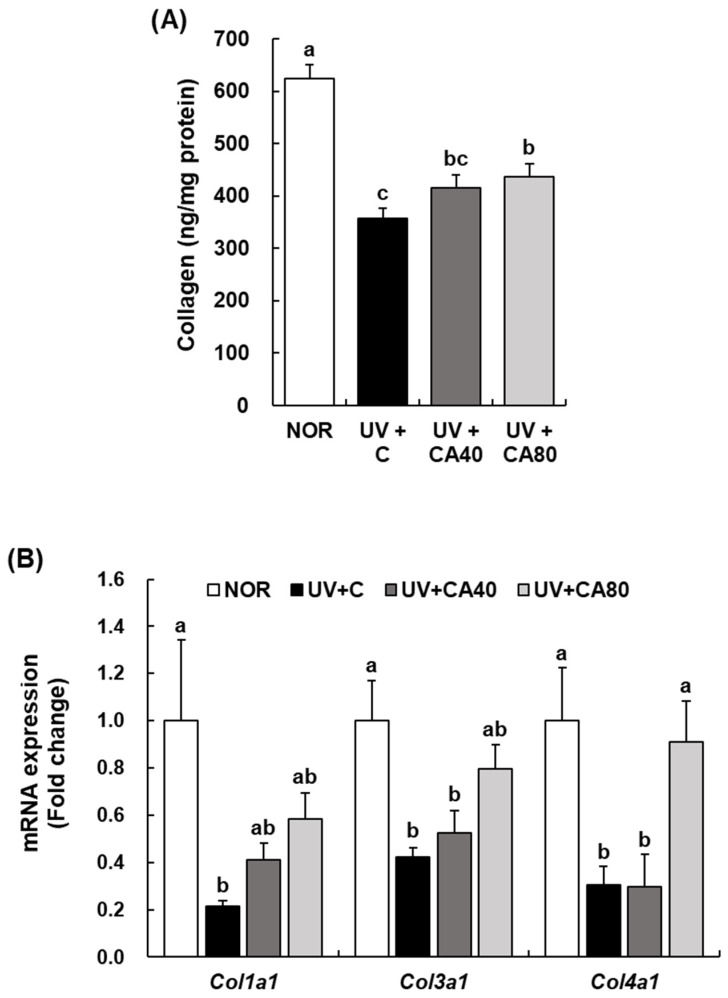
Effect of sCAE on the expression of collagen in the dorsal skin in UVB-irradiated hairless mice. All mice, except those in the NOR group, were irradiated with UVB three times weekly for 8 weeks. The mice received sCAE via oral gavage during the same period. (**A**) The dorsal skin was excised and homogenized. The content of collagen in the skin homogenate was measured using a collagen ELISA kit. (**B**) Total RNA in the dorsal skin was extracted, reverse-transcribed, and subjected to real-time PCR. The expression of each mRNA was normalized to that of *Gapdh* and is represented relative to that in the NOR group. Each bar represents the mean ± SEM (*n* = 10). Data are presented as mean ± SEM (*n* = 10 per group). Statistical analysis was performed using one-way ANOVA followed by Duncan’s multiple range test; Tukey’s honestly significant difference (HSD) test was additionally applied for conservative validation. Different letters indicate statistically significant differences at *p* < 0.05.

To evaluate the effects of sCAE on extracellular matrix (ECM) remodeling in photoaged skin, we analyzed the levels of collagen and MMPs in dorsal skin tissues. As shown in Figure 5A, UVB irradiation significantly reduced the levels of collagen compared to the NOR group (*p* < 0.001), reflecting degradation of dermal ECM. Oral administration of sCAE partially restored collagen levels, with the 80 mg/kg dose showing a significant increase compared with the UV-irradiated control, whereas the 40 mg/kg dose did not reach statistical significance (*p* > 0.05). At the gene-expression level, UVB irradiation markedly reduced *Col1a1*, *Col3a1*, and *Col4a1* mRNA expression (*p* < 0.05). sCAE administration partially restored collagen-related gene expression in a dose-dependent manner. Notably, the expression levels of *Col3a1* and *Col4a1* were significantly increased in the 80 mg/kg sCAE-treated group compared with the UV-irradiated control (*p* < 0.01 for both), whereas the 40 mg/kg dose (UV+CA40) produced partial but non-significant increases for some genes. These findings indicate that sCAE improves ECM-related gene expression in a dose-responsive manner, with the higher dose exerting more consistent restorative effects (Figure 5B).

**Figure 6:**
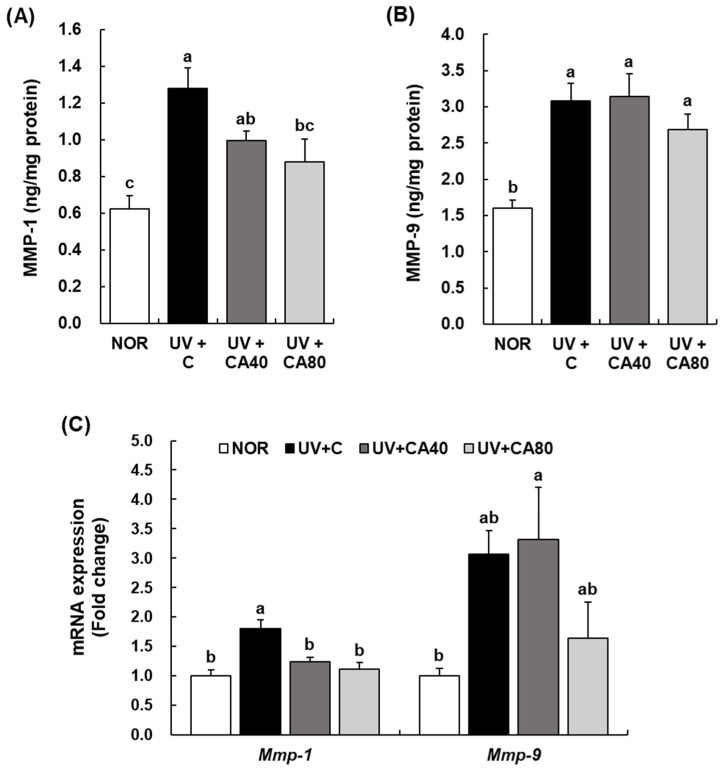
Effect of sCAE on the expression of MMP-1 and MMP-9 in the dorsal skin in UVB-irradiated hairless mice. All mice, except those in the NOR group, were irradiated with UVB three times weekly for 8 weeks. The mice received sCAE via oral gavage during the same period. (**A**,**B**) The dorsal skin was excised and homogenized. The content of MMP-1 and MMP-9 in the skin homogenate was measured using an individual ELISA kit. (**C**) Total RNA in the dorsal skin was extracted, reverse-transcribed, and subjected to real-time PCR. The expression of each mRNA was normalized to that of *Gapdh* and is represented relative to that in the NOR group. Each bar represents the mean ± SEM (*n* = 10). Data are presented as mean ± SEM (*n* = 10 per group). Statistical analysis was performed using one-way ANOVA followed by Duncan’s multiple range test; Tukey’s honestly significant difference (HSD) test was additionally applied for conservative validation. Different letters indicate statistically significant differences at *p* < 0.05.

In contrast, UVB irradiation markedly increased the expression of ECM-degrading enzymes. UVB irradiation significantly increased MMP-1 and MMP-9 protein levels compared with the normal control (*p* < 0.001 for both). Oral administration of sCAE significantly reduced MMP-1 levels in the high-dose group (80 mg/kg) relative to the UV-irradiated control under Tukey’s HSD correction (*p* < 0.05), whereas the low-dose group showed a non-significant decreasing trend (Figure 6A). For MMP-9, although sCAE treatment tended to attenuate UVB-induced increases, these differences did not reach statistical significance versus the UV-irradiated control under conservative post hoc analysis (Figure 6B). At the transcriptional level, UVB irradiation significantly increased *Mmp-1* expression compared with the NOR group (*p* < 0.001). Oral administration of sCAE significantly suppressed MMP-1 expression at both 40 and 80 mg/kg (*p* < 0.01 for both). In contrast, MMP-9 expression showed no significant reduction following sCAE treatment, although a decreasing trend was observed at 80 mg/kg (Figure 6C). In general, the UV+CA80 group demonstrated greater preservation of collagen content and more pronounced suppression of MMP expression than the UV+CA40 group, further highlighting the dose-dependent efficacy of oral sCAE in maintaining ECM integrity.

### 2.5. sCAE Downregulates Pro-Inflammatory Cytokines Expression in UVB-Irradiated Hairless Mice

**Figure 7:**
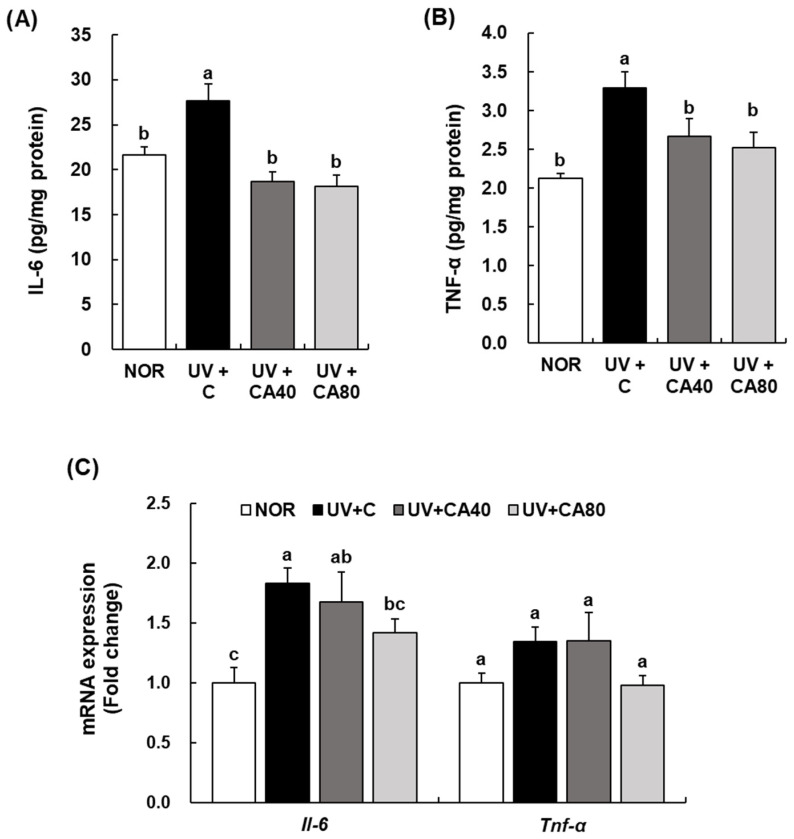
Effect of sCAE on the expression of IL-6 and TNF-α in the dorsal skin in UVB-irradiated hairless mice. All mice, except those in the NOR group, were irradiated with UVB three times weekly for 8 weeks. The mice received sCAE via oral gavage during the same period. (**A**,**B**) The dorsal skin was excised and homogenized. The content of IL-6 and TNF-α in the skin homogenate was measured using an individual ELISA kit. (**C**) Total RNA in the dorsal skin was extracted, reverse-transcribed, and subjected to real-time PCR. The expression of each mRNA was normalized to that of *Gapdh* and is represented relative to that in the NOR group. Each bar represents the mean ± SEM (*n* = 10). Data are presented as mean ± SEM (*n* = 10 per group). Statistical analysis was performed using one-way ANOVA followed by Duncan’s multiple range test; Tukey’s honestly significant difference (HSD) test was additionally applied for conservative validation. Different letters indicate statistically significant differences at *p* < 0.05.

To investigate the anti-inflammatory effects of sCAE on UVB-induced skin damage, we measured the levels of pro-inflammatory cytokines in dorsal skin tissues. As shown in Figure 7A,B, the protein levels of IL-6 and TNF-α were significantly elevated in the UV+C group compared to the NOR group (IL-6, *p* < 0.05; TNF-α, *p* < 0.001), indicating UVB-induced cutaneous inflammation.

Oral administration of sCAE significantly suppressed UVB-induced increases in pro-inflammatory cytokines. sCAE significantly attenuated UVB-induced IL-6 production at both doses relative to the UV-irradiated control under Tukey’s HSD correction (*p* < 0.001 for both). For TNF-α, a statistically significant reduction was observed only in the high-dose sCAE group (80 mg/kg) compared with the UV-irradiated control (*p* < 0.05), whereas the low-dose group showed a non-significant decreasing trend. For *Il-6* mRNA expression, UVB irradiation significantly increased transcript levels compared with the normal control (*p* < 0.01); sCAE administration at 80 mg/kg significantly attenuated UVB-induced *Il-6* mRNA expression, whereas the 40 mg/kg dose did not produce statistically significant effects. In contrast, *Tnf-α* mRNA expression did not differ significantly among groups, and all groups were therefore assigned the same statistical letter (Figure 7C). Both doses of sCAE tended to attenuate UVB-induced inflammatory cytokine production; however, these reductions did not consistently reach statistical significance under conservative post hoc testing. Notably, the higher dose showed a more pronounced decreasing trend, with cytokine levels approaching those of the NOR group.

### 2.6. sCAE Prevents Lipid Peroxidation and Enhances Antioxidant Enzyme Activities in UVB-Irradiated Hairless Mice

To examine whether sCAE protects against oxidative damage in UVB-irradiated skin, we measured MDA levels as an index of lipid peroxidation, along with the activities of key antioxidant enzymes including SOD, catalase, and GPx in skin tissue homogenates.

**Figure 8:**
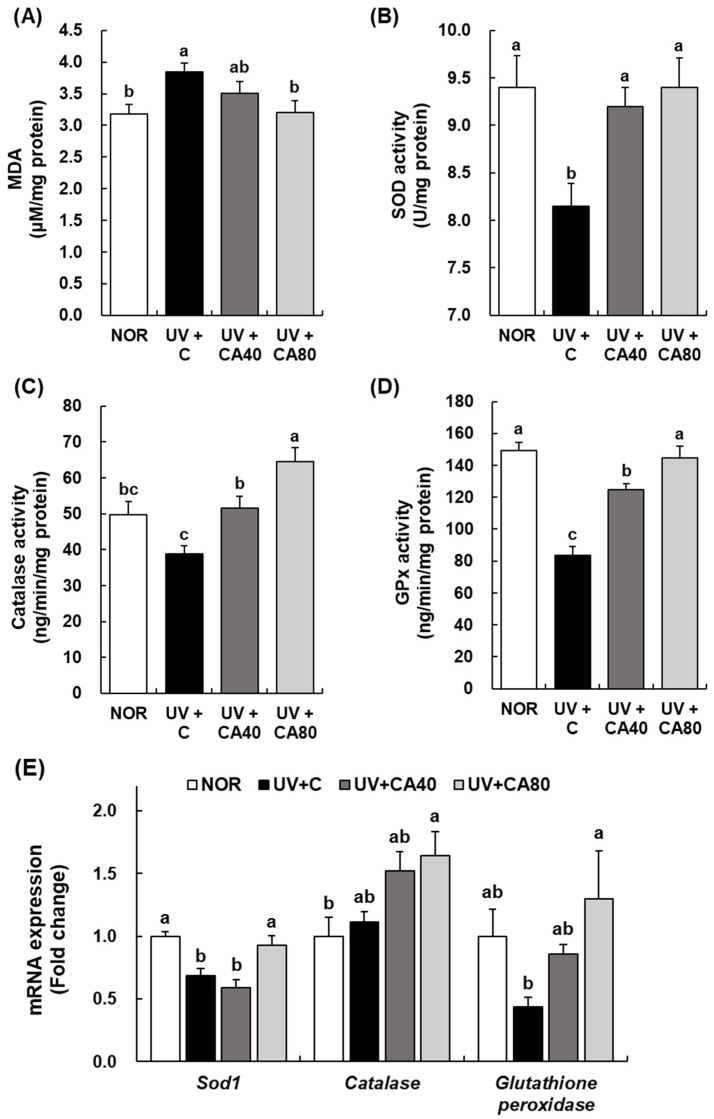
Effect of sCAE on lipid peroxidation and antioxidant enzyme activities in the dorsal skin in UVB-irradiated hairless mice. All mice, except those in the NOR group, were irradiated with UVB three times weekly for 8 weeks. The mice received sCAE via oral gavage during the same period. (**A**) The dorsal skin was excised and homogenized. The MDA content in the skin homogenate was measured using a TBARS assay kit. (**B**–**D**) The dorsal skin was excised and homogenized. The activities of SOD, catalase, glutathione peroxidase in the skin homogenate were measured the relevant assay kits. (**E**) Total RNA in the dorsal skin was extracted, reverse-transcribed, and subjected to real-time PCR. The expression of each mRNA was normalized to that of *Gapdh* and is represented relative to that in the NOR group. Each bar represents the mean ± SEM (*n* = 10). Data are presented as mean ± SEM (*n* = 10 per group). Statistical analysis was performed using one-way ANOVA followed by Duncan’s multiple range test; Tukey’s honestly significant difference (HSD) test was additionally applied for conservative validation. Different letters indicate statistically significant differences at *p* < 0.05.

As shown in Figure 8A, MDA levels were significantly elevated in the UV+C group compared to the NOR group, indicating increased lipid peroxidation due to UVB exposure (*p* < 0.05). Oral administration of sCAE reduced MDA levels in a dose-dependent manner; however, the reduction reached statistical significance only in the UV+CA80 group (*p* < 0.05 vs. UV+C), whereas the decrease observed in the UV+CA40 group was not significant. These results suggest that higher-dose sCAE more effectively mitigates UVB-induced oxidative damage.

The activities of endogenous antioxidant enzymes were differentially affected by UVB irradiation and sCAE treatment. SOD activity was significantly reduced in UVB-irradiated mice compared with NOR (*p* < 0.05). Administration of sCAE at 80 mg/kg significantly restored SOD activity relative to UV+C (*p* < 0.05), whereas the increase observed at 40 mg/kg did not reach statistical significance (Figure 8B). Catalase activity was markedly enhanced by sCAE administration (Figure 8C). The UV+CA80 group exhibited significantly higher catalase activity than UV+C (*p* < 0.001), whereas the increase in the UV+CA40 group showed only a borderline difference compared with UV+C (*p* > 0.05). Similarly, GPx activity was profoundly suppressed by UVB irradiation compared with NOR (*p* < 0.001). sCAE administration significantly restored GPx activity at both 40 mg/kg and 80 mg/kg (*p* < 0.001 vs. UV+C) (Figure 8D). At the transcriptional level, UVB irradiation significantly downregulated *Sod1* mRNA expression, whereas sCAE treatment partially restored its expression, although the changes did not reach statistical significance relative to NOR. In contrast, *catalase* and *glutathione peroxidase* mRNA expression were significantly upregulated in the UV+CA80 group compared with UV+C, while UV+CA40 induced moderate, non-significant increases (Figure 8E). These findings indicate that sCAE mitigates UVB-induced oxidative imbalance primarily through restoration of antioxidant enzyme activities, with transcriptional upregulation being more evident at the higher dose. The restorative effects of sCAE on oxidative stress markers and endogenous antioxidant enzyme activities were dose-dependent, with the UV+CA80 group showing greater reductions in lipid peroxidation and stronger recovery of antioxidant defenses compared with the UV+CA40 group.

Correlation analysis provided mechanistic insight into the coordinated regulation of skin barrier integrity, extracellular matrix (ECM) remodeling, inflammatory signaling, and oxidative stress following oral sCAE administration (Supplementary Figure S1). Transepidermal water loss (TEWL) showed strong positive correlations with MMP-1, MMP-9, and pro-inflammatory cytokines, supporting the concept that UVB-induced barrier disruption is closely linked to inflammation-driven ECM degradation. In contrast, skin hydration was positively correlated with hyaluronic acid and ceramide levels, indicating that restoration of barrier lipids and glycosaminoglycans contributes directly to improved epidermal function. Notably, collagen content was inversely correlated with MMP expression, reinforcing the central role of proteolytic ECM degradation in UVB-mediated collagen loss. In parallel, antioxidant enzyme activities (SOD, catalase, and GPx) exhibited significant negative correlations with malondialdehyde (MDA), suggesting that attenuation of oxidative stress is tightly coupled to preservation of ECM structure and barrier homeostasis. These coordinated correlations support a mechanistic framework in which oral sCAE mitigates UVB-induced photoaging by simultaneously modulating oxidative stress, inflammatory signaling, ECM degradation, and barrier dysfunction.

## 3. Discussion

In this study, we demonstrated that oral administration of sCAE effectively mitigated UVB-induced skin photoaging in Skh:HR-1 hairless mice through coordinated modulation of skin barrier function, extracellular matrix (ECM) integrity, inflammatory signaling, and oxidative stress. sCAE significantly improved skin hydration, elasticity, and wrinkle parameters, accompanied by a reduction in transepidermal water loss, indicating restoration of epidermal barrier function. These effects were associated with recovery of hyaluronic acid and ceramide levels and with upregulation of genes involved in barrier lipid and glycosaminoglycan biosynthesis, suggesting enhanced structural and functional support of the stratum corneum. At the dermal level, sCAE preserved collagen content and suppressed UVB-induced upregulation of MMP-1 and MMP-9, indicating attenuation of ECM degradation rather than simple transcriptional activation of collagen genes. This distinction is mechanistically important, as UVB-driven collagen loss is largely mediated by proteolytic degradation through MMP activation. In parallel, sCAE reduced the expression of pro-inflammatory cytokines, including TNF-α and IL-6, which are known to amplify MMP expression and disrupt ECM homeostasis under photoaging conditions. Moreover, sCAE markedly reduced lipid peroxidation while enhancing endogenous antioxidant enzyme activities, including SOD, catalase, and GPx, suggesting that suppression of oxidative stress may further contribute to inhibition of ROS-driven MMP induction and inflammatory signaling. Importantly, these protective effects exhibited a clear dose–response relationship, with the higher dose of sCAE (80 mg/kg) consistently producing outcomes that more closely approximated those of non-irradiated controls across multiple functional and molecular endpoints. This dose-dependent pattern supports the biological plausibility of sCAE efficacy and suggests that sufficient systemic exposure is required to effectively counteract the interconnected oxidative, inflammatory, and ECM-degrading pathways activated by chronic UVB exposure.

The quantification of asiaticoside in sCAE confirmed a content of 70 mg/g, supporting the consistency and reliability of the test material used in this study. Asiaticoside is a well-characterized pentacyclic triterpenoid saponin recognized as one of the principal bioactive constituents responsible for the pharmacological actions of *C. asiatica*, including its antioxidant, anti-inflammatory, and skin-regenerative properties [25,26]. Similar HPLC-based standardization approaches have been reported in prior studies. Lu et al. [22] reported asiaticoside-induced collagen synthesis in human dermal fibroblasts and highlighted the importance of dose-controlled administration of purified extracts in achieving reproducible biological outcomes. Likewise, Jiang et al. [20], quantified asiaticoside and demonstrated its anti-photoaging potential through TGF-β1/Smad pathway modulation in UV-exposed cells. Compared with these previous reports, the current study provides an essential extension by confirming both the chemical composition and the biological efficacy of a standardized oral formulation of *C. asiatica* extract in an in vivo photoaging model. The use of an extract standardized to a specific asiaticoside concentration not only enhances the reproducibility of results but also facilitates the translation of preclinical findings into potential human applications. These findings support the rationale for using standardized phytochemical-rich formulations in future nutraceutical development aimed at skin health and anti-aging applications.

Oral administration of sCAE significantly alleviated UVB-induced photoaging in hairless mice by reducing wrinkle formation, restoring skin hydration and elasticity, and improving barrier function. These findings are consistent with earlier reports demonstrating the skin-protective effects of *C. asiatica*. Legiawati et al. [24] reported that combined oral and topical administration of *C. asiatica* improved skin hydration and reduced pro-aging markers in patients with type 2 diabetes and dry skin. Similarly, Sunikumar et al. [11], found that topical application of *C. asiatica* extracts enhanced wound healing and improved skin architecture in rodent models. Unlike previous studies, the present investigation employed a well-characterized, standardized extract containing 70 mg/g of asiaticoside. It evaluated its efficacy through oral delivery in a UVB-induced photoaging mouse model. Notably, quantitative assessments of wrinkle morphology using skin replicas, along with biophysical measurements such as TEWL and elasticity, provided objective evidence of sCAE’s anti-photoaging effects. These results underscore the reliability and translational potential of using sCAE as a dietary supplement to promote skin health. Taken together, our findings suggest that *C. asiatica* is not only a traditional wound-healing agent but also a promising oral nutricosmetic candidate capable of mitigating extrinsic skin aging. Compared to topical agents, oral administration offers the advantage of systemic absorption, potentially providing broader and longer-lasting protection against photoaging-related damage.

Disruption of the skin barrier and reduction in essential moisturizing factors such as hyaluronic acid and ceramides are hallmark features of UVB-induced photoaging [27]. In this study, oral administration of sCAE significantly restored the levels of hyaluronic acid and ceramide in UVB-irradiated hairless mice, and upregulated the expression of related biosynthetic genes (*Has1*, *Has3*, and *Sptlc2*), suggesting its beneficial role in improving skin hydration and lipid barrier homeostasis. These findings are consistent with previous reports demonstrating the moisturizing and barrier-reinforcing effects of *C. asiatica*. Shen et al. [28], showed that madecassoside, a major triterpenoid of *C. asiatica*, upregulated HAS1–3 and AQP3 in human keratinocytes, increasing hyaluronan content and supporting moisture retention. In line with these cellular findings, a randomized assessor-blinded clinical study reported improved skin hydration and reduced TEWL after topical use of a formulation containing *C. asiatica*–derived actives [29]. Compared to those studies, our work provides direct in vivo evidence that orally administered, standardized *C. asiatica* extract can restore both the biochemical (ELISA) and transcriptional (qPCR) profiles of key hydration-related markers in UVB-compromised skin. Notably, the dose-dependent response observed with sCAE administration underscores its potential for tailored therapeutic strategies. These results suggest that sCAE may serve as a promising oral intervention for reinforcing the skin’s natural moisturizing factors and ceramide biosynthesis, contributing to the prevention of UVB-induced barrier disruption and dryness. As such, sCAE holds substantial potential as a nutraceutical ingredient in skin health formulations targeting extrinsic aging and compromised barrier function.

Photoaging is characterized by collagen degradation and ECM disorganization, primarily mediated by increased MMPs activity and reduced collagen biosynthesis [30]. In the present study, oral administration of sCAE significantly upregulated the expression of collagen, while downregulating MMP-1 and MMP-9 at both the protein and mRNA levels in UVB-irradiated hairless mice. These findings suggest that sCAE protects dermal structure by promoting ECM remodeling and suppressing UVB-induced collagen degradation. Notably, a divergence between collagen protein content and collagen-related gene expression was observed, prompting further consideration of ECM regulatory mechanisms. The apparent discrepancy between collagen protein content and collagen-related mRNA expression observed in Figure 5 likely reflects the complex, multi-level regulation of ECM homeostasis. Collagen protein accumulation represents the net balance between synthesis and degradation and is subject to temporal delays relative to transcriptional changes. UVB-induced collagen loss is primarily driven by MMP-mediated degradation rather than transcriptional suppression alone. Accordingly, partial restoration of collagen protein content following sCAE administration may occur through inhibition of ECM degradation, even when collagen mRNA expression remains incompletely recovered. In addition, post-transcriptional and post-translational regulatory mechanisms further contribute to differences between mRNA expression and protein abundance. Similar discrepancies between collagen mRNA and protein levels have been reported in other UVB-induced photoaging models. These results are consistent with previous studies reporting the collagen-stimulating and MMP-inhibitory properties of *C. asiatica* and its active compounds. Kencana et al. [31] conducted a comprehensive bioinformatics and molecular docking study that predicted asiaticoside exhibits strong binding affinity to key skin-aging mediators such as MMP-9, suggesting it may directly inhibit ECM degradation pathways. Furthermore, Rahmawati et al. [32] also observed that oral and topical *C. asiatica* extract reduced UVB-induced dermal MMP-1 activity and preserved skin structure in hairless rats. Compared to these prior studies, our findings provide more comprehensive in vivo evidence showing that oral supplementation with a chemically standardized extract (70 mg/g asiaticoside) results in a dual action—enhancing collagen synthesis while inhibiting ECM-degrading enzymes. The dose-dependent effects observed further support the reproducibility and potential clinical relevance of sCAE as a nutraceutical ingredient. These results highlight the therapeutic potential of sCAE in maintaining dermal ECM integrity under UVB-induced oxidative stress conditions. By simultaneously targeting both synthesis and degradation pathways, sCAE may serve as a multifunctional anti-photoaging agent capable of preserving skin firmness and structural integrity.

Chronic exposure to UVB radiation induces inflammation-mediated skin aging by upregulating pro-inflammatory cytokines such as TNF-α and IL-6, which accelerate matrix degradation and impair skin homeostasis [33]. In this study, oral administration of sCAE attenuated UVB-induced increases in both the protein and mRNA levels of TNF-α and IL-6, indicating its potent anti-inflammatory action in photoaged skin. These findings are consistent with previous studies demonstrating the anti-inflammatory properties of *C. asiatica* and its triterpenoid constituents. Cho et al. [14] reported that *C. asiatica* extract inhibited inflammatory responses in LPS-stimulated macrophages by downregulating the IRAK1-TAK1 signaling axis. Moreover, Jiang et al. [20] showed that asiaticoside suppressed UV-induced expression of TNF-α and IL-6 in dermal fibroblasts via the TGF-β1/Smad pathway, thereby reducing oxidative and inflammatory stress. In vivo, Rahmawati et al. [32] observed that *C. asiatica* extract lowered MMP-1 and inflammatory cytokines in UVB-irradiated skin tissues, further supporting its systemic anti-inflammatory potential. Compared to those reports, our study uniquely confirms the anti-inflammatory efficacy of oral sCAE in an animal model of UVB-induced photoaging, with apparent dose-dependent effects. The simultaneous downregulation of both cytokine protein levels and gene expression enhances the reliability of the evidence and strengthens the rationale for sCAE as a dietary anti-photoaging strategy. Taken together, these results highlight that the photoprotective effects of sCAE are not only attributed to antioxidant and ECM-preserving actions, but also to its ability to suppress chronic inflammation—an essential driver of extrinsic skin aging. These properties support the utility of sCAE as a promising nutraceutical intervention for inflammatory skin conditions aggravated by UV exposure.

Oxidative stress is a major contributor to UVB-induced photoaging, primarily through the accumulation of reactive oxygen species that trigger lipid peroxidation and impair the skin’s antioxidant defense systems [34]. In the present study, oral administration of sCAE reduced MDA levels—a well-established marker of lipid peroxidation-while concurrently enhancing the activities of endogenous antioxidant enzymes such as SOD, catalase, and GPx. These results indicate that sCAE mitigates UVB-induced oxidative damage and supports redox homeostasis in the skin. These findings are consistent with earlier reports on the antioxidant properties of *C. asiatica* [35]. Jayashree et al. [13] demonstrated that *C. asiatica* extract reduced MDA levels and increased SOD and CAT activities in lymphoma-bearing mice. Furthermore, Rachpirom et al. [19] confirmed that a standardized *C. asiatica* extract improved antioxidant capacity and reduced oxidative biomarkers in murine wound models. Compared to these studies, our findings extend the evidence base by demonstrating that orally administered, asiaticoside-standardized sCAE not only reduces oxidative stress markers but also restores key enzymatic antioxidant defenses in vivo under UVB-induced stress conditions. Taken together, these results suggest that the anti-photoaging effects of sCAE are in part mediated by its ability to neutralize oxidative stress and reinforce the skin’s endogenous antioxidant network. Such dual action supports the development of sCAE as a nutritional antioxidant agent for skin health, particularly in populations chronically exposed to environmental oxidative stressors such as UV radiation.

To further integrate these findings and clarify their mechanistic interrelationships, correlation analysis was performed across key skin biophysical and molecular parameters. The correlation analysis provides additional mechanistic insight into the coordinated effects of oral sCAE on UVB-induced skin photoaging. The positive associations between TEWL and MMPs or inflammatory cytokines, together with the inverse relationships between collagen content and MMP expression, support the role of ECM degradation in barrier dysfunction. Moreover, the strong negative correlations between antioxidant enzyme activities and MDA suggest that oxidative stress modulation is closely linked to the preservation of skin structure and function. These findings reinforce the integrated antioxidant, anti-inflammatory, and ECM-preserving actions of sCAE.

Although upstream signaling pathways were not directly examined in the present study, extensive evidence from previous studies provides mechanistic context for the observed ECM-protective effects of oral sCAE. UVB-induced collagen degradation is primarily mediated through activation of MAPK (ERK, JNK, p38) and AP-1 signaling, leading to upregulation of MMPs, while suppression of TGF-β/Smad signaling impairs collagen synthesis. Asiaticoside and related triterpenoids from *C. asiatica* have been reported to inhibit MAPK and NF-κB activation, thereby reducing MMP expression and inflammatory cytokine production, while simultaneously activating the TGF-β1/Smad pathway to promote collagen synthesis in dermal fibroblasts. In addition, asiaticoside has been shown to enhance antioxidant defenses via Nrf2-dependent pathways, which may indirectly preserve ECM integrity by attenuating ROS-driven MMP induction. The coordinated modulation of these pathways provides a plausible mechanistic framework explaining the simultaneous restoration of collagen, suppression of MMPs, reduction in inflammatory cytokines, and enhancement of antioxidant capacity observed in the present study.

This study possesses several notable strengths. First, it utilized a chemically standardized *C. asiatica* extract (sCAE) containing 70 mg/g of asiaticoside, ensuring reproducibility and scientific reliability. Second, the study employed a well-established UVB-induced photoaging mouse model and comprehensively evaluated the protective effects of sCAE using macroscopic, biophysical, histological, biochemical, and molecular endpoints. The inclusion of two dose groups allowed for dose–response analysis, further reinforcing the validity of the findings. Moreover, the study provided mechanistic insights by demonstrating that sCAE exerts its anti-photoaging effects through multiple pathways, including enhancement of collagen synthesis, suppression of MMPs, attenuation of inflammatory cytokines, and restoration of antioxidant defense. Lastly, by focusing on oral administration, the study highlights the potential of sCAE as a nutricosmetic or dietary intervention for skin health and photoaging prevention.

While the present study provides strong evidence for the anti-photoaging effects of sCAE in a UVB-induced hairless mouse model, several limitations should be acknowledged. First, the experiments were conducted exclusively in male Skh:HR-1 mice, which may limit the generalizability of the findings to females, other age groups, or humans. Sex hormones, particularly estrogen, are known to modulate skin barrier function, collagen synthesis, inflammatory responses, and antioxidant capacity, and may influence both susceptibility to UVB-induced photoaging and responsiveness to nutraceutical interventions. Although the use of male mice was intended to reduce variability associated with estrous cycle–dependent hormonal fluctuations, future studies incorporating female animals and sex-specific analyses will be essential to determine whether similar efficacy and mechanisms are observed across sexes. Second, while multiple molecular and biochemical biomarkers were quantified, upstream signaling pathways implicated in ECM remodeling and oxidative stress regulation, such as MAPK, NF-κB, and Nrf2 signaling, were not directly examined. Further mechanistic studies employing pathway-specific inhibitors or genetic models are warranted to clarify the precise molecular targets of sCAE. Third, the bioavailability and metabolic fate of asiaticoside following oral administration were not assessed, limiting direct linkage between systemic exposure and observed efficacy. In addition, although the extract was standardized for asiaticoside content, the potential synergistic contributions of other bioactive triterpenoids, including madecassoside and asiatic acid, were not independently evaluated. Finally, from a statistical perspective, although Duncan’s multiple range test was used as the primary post hoc method due to its sensitivity in nutritional and preclinical studies, certain protein-level parameters—such as collagen content and MMP-9 expression—did not retain statistical significance under the more conservative Tukey’s HSD correction, likely reflecting biological variability inherent in in vivo protein measurements and stringent control of family-wise error. Importantly, the overall directional trends remained consistent across statistical approaches, and key functional and mechanistic outcomes—including skin barrier parameters, inflammatory cytokines, and antioxidant enzyme activities—were robust under both methods. Future studies incorporating larger sample sizes, additional dose levels, longitudinal designs, topical comparison arms, and ultimately clinical trials will be necessary to fully validate the safety, efficacy, and mechanisms of sCAE as an anti-photoaging nutraceutical.

## 4. Materials and Methods

### 4.1. Preparation of sCAE

Dried *C. asiatica* leaves were extracted twice with 70% (*v*/*v*) ethanol at 80 °C for 6 h. The resulting extract was filtered and concentrated under reduced pressure at 65 °C to 20–25° Brix. The concentrate was then spray-dried to obtain a powder as sCAE (InnerCica^TM^, 3H LABS Co. Ltd., Goyang, Republic of Korea), which was stored under light-protected, sealed conditions until use.

### 4.2. Quantification of Asiaticoside in sCAE

sCAE was analyzed by high-performance liquid chromatography (HPLC) using the Waters e2695 system equipped with a photodiode array detector (Waters Co., Ltd., Milford, MA, USA). Separations were performed on a Cadenza C18 column (250 × 4.6 mm, 3 µm; Imtakt, Portland, OR, USA) maintained at 40 °C, with a flow rate of 1.0 mL/min. The mobile phases were distilled water (A) and acetonitrile (B). A linear gradient was applied as follows (A/B, *v*/*v*): 0 min, 90/10; 10 min, 80/20; 40 min, 73/27; 45 min, 80/20; 51 min, 20/80; and 55 min, 90/10. The injection volume was 10 µL, and the UV wavelength was set at 206 nm. Asiaticoside used as a reference standard, was purchased from Chengdu Biopurify Phytochemicals, Ltd. (Chengdu, China).

### 4.3. Ethical Statement and Animal Care

All animal experimental protocols were approved by the Institutional Animal Care and Use Committee of Sahmyook University (approved number: SYUIACUC 2024-011). The care and use of the animals followed established guidelines for the care and use of laboratory animals.

Five-week-old male Skh:HR-1 hairless mice were procured from Raonbio Co., Ltd. (Yongin, Republic of Korea). The mice were maintained under controlled conditions (23 ± 3 °C, 50 ± 10% relative humidity, and 12 h light/dark cycles) and provided with a commercial rodent diet and tap water ad libitum.

To minimize potential confounding factors, all animals were housed within the same room under identical environmental conditions, and cages were randomly assigned to rack positions to eliminate location-related effects. All treatments, including oral gavage and UVB irradiation, were administered at the same time each day to reduce variability associated with circadian rhythms. Measurements such as body weight, food intake, and skin-related indices were obtained in a consistent order across all groups to avoid systematic measurement bias. Throughout the experimental period, animals were closely monitored for general health indicators—including body weight, food consumption, grooming, posture, and any abnormal behaviors. No adverse effects related to sCAE administration were observed. Aside from the factors explicitly monitored and controlled, no additional confounding variables were identified during the study period.

All animals were sourced as specific pathogen-free (SPF) and had no history of prior experimental procedures before arrival. Skh:HR-1 hairless mice are immunocompetent despite lacking pelage hair, and no abnormalities in health or immune status were observed during the acclimation period.

### 4.4. Experiment Design, UVB Irradiation, and Treatment

After a one-week acclimation period, a total of 40 Skh:HR-1 hairless mice were used in this study. Animals were randomly assigned to experimental groups using a computer-generated random allocation sequence to minimize selection bias. In addition, cage positions were randomized throughout the study period to avoid potential environmental confounding factors, such as rack location or differential light exposure. Each mouse served as an independent experimental unit for all outcome measurements and statistical analyses. The four groups consisted of: (i) normal control group (without UVB irradiation, vehicle-treated group, NOR); (ii) UVB-irradiated control group (UVB irradiation, vehicle-treated group, UV+C); (iii) UVB-irradiated and 40 mg/kg body weight (BW)/day sCAE-treated group, UV+CA40); and (iv) UVB-irradiated and 80 mg/kg BW/day sCAE-treated group, UV+CA80). A pre-specified study protocol, including the research question, experimental design, dosing plan, UVB irradiation schedule, outcome measures, and statistical analysis approach, was prepared before conducting the experiment and approved within the IACUC application (SYUIACUC 2024-011). No deviations from the protocol occurred, and the study was not registered in a public repository, consistent with current norms for preclinical animal research.

The primary outcome measure of this study was the improvement of UVB-induced photoaging severity, assessed by quantitative wrinkle parameters derived from dorsal skin replicas (total roughness, average roughness, and maximum roughness). Secondary outcome measures included skin hydration, transepidermal water loss (TEWL), elasticity indices, epidermal thickness, antioxidant enzyme activities, ECM-related markers (collagen, MMP-1, MMP-9), pro-inflammatory cytokines, and barrier components (hyaluronic acid and ceramide).

The sample size was determined a priori based on power considerations derived from previously published UVB-induced photoaging studies using Skh:HR-1 hairless mice. Prior studies evaluating key endpoints such as epidermal thickness, wrinkle parameters, and transepidermal water loss have reported large effect sizes (Cohen’s d ≈ 1.1–1.3). Based on these effect sizes, a group size of 8–10 animals was estimated to provide at least 80% statistical power at a significance level of α = 0.05. Considering the 8-week UVB irradiation protocol and potential inter-individual variability, a conservative sample size of 10 mice per group was selected to ensure statistical robustness. No animals were excluded from the analysis. No predefined inclusion or exclusion criteria were established for animals or individual data points, and all animals assigned to each group completed the study. All data collected were retained for statistical analysis without omission.

UVB irradiation was performed using a UVB-specific irradiator (UV1-100, BoTech, Gunpo, Republic of Korea) equipped with a GL20SE lamp (Sankyo Denki, Yokohama, Japan) emitting wavelengths of 280–325 nm (peak 306 nm). All mice, except those in the NOR group, were irradiated three times per week for 8 weeks following a progressively increasing UVB dose schedule: 60 mJ/cm^2^ (weeks 1–2), 120 mJ/cm^2^ (weeks 3–4), 180 mJ/cm^2^ (weeks 5–6), and 240 mJ/cm^2^ (weeks 7–8). During the same 8-week period, mice received daily oral gavage of vehicle (distilled water) or sCAE (40 or 80 mg/kg BW/day).

Body weight and food intake were measured weekly throughout the study to monitor growth, nutritional status, and potential treatment-related effects. Upon completion of the 8-week treatment period, mice were fasted overnight for 16 h to standardize metabolic conditions before sample collection. To minimize stress and ensure humane handling, mice were anesthetized with tribromoethanol diluted in tertiary amyl alcohol (250 mg/kg BW, intraperitoneal injection). All skin physiological parameters, including transepidermal water loss, hydration index, elasticity, and wrinkle analysis using skin replicas, were assessed at a single standardized terminal time point following completion of the 8-week UVB irradiation and oral treatment period. All measurements were performed prior to euthanasia under controlled environmental conditions and at the same time of day for all animals to minimize circadian and environmental variability. Following anesthesia, various biophysical skin indices were measured. Mice were then euthanized by cervical dislocation, and dorsal skin tissues were promptly harvested for histological, biochemical, and molecular analyses.

To reduce measurement and observer bias, personnel responsible for UVB irradiation and oral gavage were aware of group assignments due to procedural requirements. However, all outcome assessments—including macroscopic skin evaluation, wrinkle replica analysis, transepidermal water loss, hydration and elasticity measurements, histological quantification, ELISA-based biochemical assays, enzymatic activity measurements, and quantitative real-time PCR analyses—were performed by investigators who remained blinded to group allocation until all data collection and analyses were completed.

### 4.5. Evaluation of the Indices Reflecting the Skin Condition

Skin hydration index, transepidermal water loss (TEWL), and elasticity index were estimated using Corneometer^®^ CM825, Tewameter^®^ TM300, and Cutometer^®^ MPA580 (CourageKhazaka Electronic GmbH, Köln, Germany), respectively. To evaluate wrinkle condition, skin replicas were cast on the dorsal skin surface of mice using SILFLO (Flexico Developments Ltd., Tokyo, Japan) and measured with a Visionmeter SV600 (Courage-Khazaka Electronic GmbH, Köln, Germany). The topography of the skin surface was then analyzed to determine its total roughness (the distance between the highest peak and the lowest value), average roughness (the average of the 5 maximum distances), and maximum roughness (the largest value of the 5 maximum distances.

### 4.6. Histological Examination

Dorsal skin tissues were fixed in 4% paraformaldehyde immediately after removal. The fixed skin tissues were embedded in paraffin, sectioned to a thickness of 5 µm, deparaffinized with xylene, and rehydrated in a series of decreasing alcohols, culminating in distilled water. The tissue sections were stained with hematoxylin and eosin (H&E). The histological changes were blindly examined and photographed at 100× magnification under a light microscope (ECLIPSE Ts2, Nikon, Tokyo, Japan). The thickness of the epidermal layer was measured.

### 4.7. Enzyme-Linked Immunosorbent Assay (ELISA)

Dorsal skin tissues were homogenized in phosphate-buffered saline and centrifuged at 5000 rpm for 10 min. The supernatant was collected and subjected to ELISA. The protein content of the supernatant was measured using a bicinchoninic acid (BCA) protein assay kit (Thermo Scientific, Rockford, IL, USA). The levels of hyaluronic acid (R&D Systems, Minneapolis, MN, USA), ceramide (MyBioSource, San Diego, CA, USA), collagen (Abcam, Cambridge, UK), matrix metalloproteinase (MMP)-1 (MyBioSource), MMP-9 (R&D Systems), interleukin (IL)-6 (R&D Systems), and tumor necrosis factor (TNF)-α (R&D Systems) in skin homogenates were measured using the relevant ELISA kits according to the manufacturer’s instructions.

### 4.8. Measurement of Lipid Peroxidation and Antioxidant Enzyme Activities

The dorsal skin homogenates were prepared, and the protein content of each was measured as described above. To evaluate lipid peroxidation in skin homogenates, the malondialdehyde (MDA) content was determined using a thiobarbituric acid reactive substance (TBARS) assay kit (Cayman Chemical, Ann Arbor, MI, USA). The activities of superoxide dismutase (SOD, Cayman Chemical), catalase (Cayman Chemical), and glutathione peroxidase (GPx, Cayman Chemical) in skin homogenates were measured using the relevant assay kits, according to the manufacturer’s instructions.

### 4.9. Quantitative Real-Time Reverse Transcription Polymerase Chain Reaction (RT-PCR)

The total RNA was extracted from dorsal skin using a Trizol^®^ reagent (Invitrogen Life Technologies, Carlsbad, CA, USA). Single-strand complementary DNA was synthesized using a HyperScript^TM^ RT Master Mix kit (GeneAll Biotechnology, Seoul, Republic of Korea). Real-time PCR was performed using the specific primers, as listed in Table 1, a QuntiNova^TM^ SYBR Green PCR kit (Qiagen, Valencia, CA, USA), and real-time thermal cycler (CFX96^TM^ Real-time system, Bio-Rad Laboratories, Hercules, CA, USA). All procedures were performed following the manufacturer’s instructions. The results were analyzed using CFX Maestro^TM^ Software (version 2.2, Bio-Rad Laboratories). The relative mRNA expression levels of target genes were normalized to those of glyceraldehyde 3-phosphate dehydrogenase (*Gapdh*).

### 4.10. Statistical Analysis

All data are presented as the mean ± standard error of the mean (SEM). Statistical analysis was performed with One-way analysis of variance followed by Duncan’s multiple comparisons test using the SPSS software (IBM SPSS Statistics 26, IBM Corp., Armonk, NY, USA). Prior to one-way analysis of variance (ANOVA), the assumptions of normality and homogeneity of variances were evaluated. Normality of data distribution was assessed using the Shapiro–Wilk test, which is appropriate for small-to-moderate sample sizes (n = 10 per group). Homogeneity of variances was examined using Levene’s test. All datasets satisfied the assumptions of normal distribution and equal variance; therefore, no data transformations were required prior to statistical analysis. Post hoc comparisons were initially performed using Duncan’s multiple range test, which is widely applied in nutritional science and preclinical functional food studies, particularly in experiments with equal group sizes and multiple dose groups where sensitivity in detecting intergroup differences is required. To further strengthen the robustness of the statistical conclusions, major datasets were additionally reanalyzed using Tukey’s honestly significant difference (HSD) test, a more conservative post hoc method commonly used in biomedical research. In all bar graphs, different superscript letters above the bars indicate statistically significant differences among groups at *p* < 0.05, as determined by Duncan’s multiple range test following one-way ANOVA. Unless otherwise stated, the sample size for each group was *n* = 10, and these details are explicitly included in the corresponding figure legends to ensure transparency and reproducibility.

## 5. Conclusions

This study demonstrated that oral administration of sCAE, containing 70 mg/g of asiaticoside, effectively attenuates UVB-induced skin photoaging in Skh:HR-1 hairless mice. sCAE significantly improved skin hydration, elasticity, and wrinkle morphology, while restoring barrier components such as hyaluronic acid and ceramide. Furthermore, sCAE enhanced collagen synthesis, suppressed matrix-degrading enzymes (MMP-1, MMP-9), downregulated pro-inflammatory cytokines (TNF-α, IL-6), and mitigated oxidative stress by reducing lipid peroxidation and upregulating antioxidant enzymes (SOD, catalase, GPx). These multifaceted effects collectively suggest that sCAE exerts strong protective effects against UVB-induced photoaging through antioxidant, anti-inflammatory, and extracellular matrix-preserving mechanisms. Taken together, these findings support the potential of sCAE as a promising oral nutraceutical intervention for promoting skin health and preventing photoaging. Future clinical studies are warranted to validate its efficacy in humans and to further elucidate its molecular mechanisms of action in diverse skin-aging contexts.

## Figures and Tables

**Table 1:** Primer sequences used in this study.

Target Gene	Forward Primer (5′-3′)	Reverse Primer (5′-3′)
*Aqp3*	AACTTGGCTTTTGGCTTCGC	CTTGATCCAGGGCTCTCGTG
*Catalase*	ACGAGGAGGAGAGGAAACGC	AAGCTGAGCGTCCTTCAGGT
*Cers3*	CACGCAGAACGTCCAGGAT	TGTTGGTCCCAAGGCCATTC
*Col1a1*	CCTCAGGGTATTGCTGGACAAC	CAGAAGGACCTTGTTTGCCAGG
*Col3a1*	ACCAAGGCTGCAAGATGGATG	ACAGTCATGGGGCTGGCATTTA
*Col4a1*	TCCGGGAGTACCAGGATATGGA	GGCTCCCCCTTTCTCCTTTTTC
*Flg2*	ATCAGTCTTGCCGTACCCAGTC	CTGACCTTCTGAGACACACCCAT
*Glutathione peroxidase*	CGGTTTCCCGTGCAATCAGT	GGTCGGACGTACTTGAGGGAA
*Has1*	GTGCGAGTGTTGGATGAAGACC	CCACATTGAAGGCTACCCAGTATC
*Has3*	CAAAGTAGGAGCTGGAACCGGT	AAACAAGGAAATCGGCAGCCAG
*Hyal1*	TGCCCGTAATGCCCTACGT	AGTTCCTCCAGGGGCAGAAG
*Il-6*	TACCACTTCACAAGTCGGAGGC	TGGTACTCCAGAAGACCAGAGG
*Mmp-1*	TTGCCCAGAGAAAAGCTTCAGC	TAGCAGCCCAGAGAAGCAACA
*Mmp-9*	ACGTGGGTCGATTCCAAACCT	GTCTCGCGGCAAGTCTTCAG
*Sptlc2*	GACCCACCTGAACAGTGCTC	AATCTTCCTAGCCCAGGAGGAG
*Sod1*	GGTGAACCAGTTGTGTTGTCAGG	ATGAGGTCCTGCACTGGTACAG
*Tnf-α*	GGTGCCTATGTCTCAGCCTCTT	GCCATAGAACTGATGAGAGGGAG
*Gapdh*	TGTGTCCGTCGTGGATCTGA	TTGCTGTTGAAGTCGCAGGAG

## Data Availability

The original contributions presented in this study are included in the article/Supplementary Materials. Further inquiries can be directed to the corresponding author.

## Supplementary Materials

The following supporting information can be downloaded at: https://www.mdpi.com/article/10.3390/ijms27010204/s1.
