# Supplementary material for: Oral Centella asiatica Extract Attenuates UVB-Induced Skin Photoaging via Antioxidant, Anti-Inflammatory, and Extracellular Matrix-Preserving Effects in Hairless Mice
Source: Int J Mol Sci. 2025 Dec 24;27(1):204. doi: 10.3390/ijms27010204 (PMC12785454; doi:10.3390/ijms27010204)
Supplement: Supplementary file 1 [file ijms-27-00204-s001.zip › ijms-4040027-supplementary.pdf]

Supplementary Table S1. Summary of quantitative outcomes and post hoc comparisons

A. Skin barrier and biophysical parameters

**Variable**	**NOR**	**UV+C**	**UV+CA40**	**UV+CA80**	**Duncan**	**Tukey vs UV+C (*p*-value)**
TEWL (g/h/m²)	7.1 ± 1.1	34.8 ± 3.0	29.8 ± 3.3	26.8 ± 2.6	c / a / ab / b	0.0516 / **0.0146**
Hydration index	28.9 ± 2.1	20.5 ± 1.6	20.3 ± 1.8	29.0 ± 2.7	a / b / b / a	0.8450 / **0.0180**

B. Barrier lipids–related markers

**Variable**	**NOR**	**UV+C**	**UV+CA40**	**UV+CA80**	**Duncan letters**	**Tukey vs. UV+C (*p*-value)**
Hyaluronic acid (μg/mg protein)	11.87 ± 0.51	7.84 ± 0.47	9.13 ± 0.78	10.23 ± 0.68	a / c / bc / ab	0.1733 / **0.0269**
Ceramide (ng/mg protein)	1.51 ± 0.16	1.11 ± 0.07	1.40 ± 0.13	1.53 ± 0.14	a / b / ab / a	0.0738 / **0.0125**

C. Extracellular matrix (ECM)–related markers

**Variable**	**NOR**	**UV+C**	**UV+CA40**	**UV+CA80**	**Duncan**	**Tukey vs UV+C (*p*-value)**
**Collagen (ng/mg protein)**	624.9 ± 26.5	357.6 ± 19.1	415.2 ± 25.4	437.5 ± 24.5	a / c / bc / b	0.342 / 0.106 †
MMP-1 (ng/mg protein)	0.62 ± 0.07	1.28 ± 0.11	0.99 ± 0.05	0.88 ± 0.12	c / a / b / bc	**<0.001 / <0.001**
MMP-9 (ng/mg protein)	1.60 ± 0.12	3.08 ± 0.24	3.14 ± 0.31	2.69 ± 0.21	b / a / a / a	0.912 / 0.065 †

D. Inflammatory cytokines

**Variable**	**NOR**	**UV+C**	**UV+CA40**	**UV+CA80**	**Duncan**	**Tukey vs UV+C (*p*-value)**
IL-6 (pg/mg protein)	21.6 ± 1.0	27.6 ± 1.9	18.7 ± 1.1	18.2 ± 1.2	b / a / b / b	**<0.001 / <0.001**
TNF-α (pg/mg protein)	2.13 ± 0.06	3.29 ± 0.20	2.67 ± 0.23	2.52 ± 0.20	b / a / b / b	**0.003 / 0.001**

E. Oxidative stress and antioxidant defense

**Variable**	**NOR**	**UV+C**	**UV+CA40**	**UV+CA80**	**Duncan**	**Tukey vs UV+C (*p*-value)**
MDA (μM/mg protein)	3.18 ± 0.15	3.85 ± 0.14	3.51 ± 0.19	3.20 ± 0.19	b / a / ab / b	0.118 / **0.021**
SOD (U/mg protein)	9.40 ± 0.33	8.15 ± 0.24	9.20 ± 0.21	9.40 ± 0.31	a / b / a / a	0.0516 / **0.0146**
Catalase (nmol/min/mg)	49.7 ± 3.7	38.7 ± 2.4	51.6 ± 3.4	64.6 ± 3.9	a / b / a / a	0.0507 / **<0.0001**
GPx (nmol/min/mg)	149.4 ± 5.1	83.7 ± 5.4	124.7 ± 3.5	144.6 ± 7.2	a / c / b / a	**<0.0001 / <0.0001**

- Data are presented as mean ± SEM.
- Different letters indicate statistically significant differences at $p < 0.05$ based on one-way ANOVA followed by Duncan's multiple range test.
- Tukey's honestly significant difference (HSD) test was used for conservative confirmation of pairwise comparisons versus UV+C.
- † Indicates trends that did not reach statistical significance under Tukey's correction.

Supplementary Figure S1. Pearson Correlation Matrix

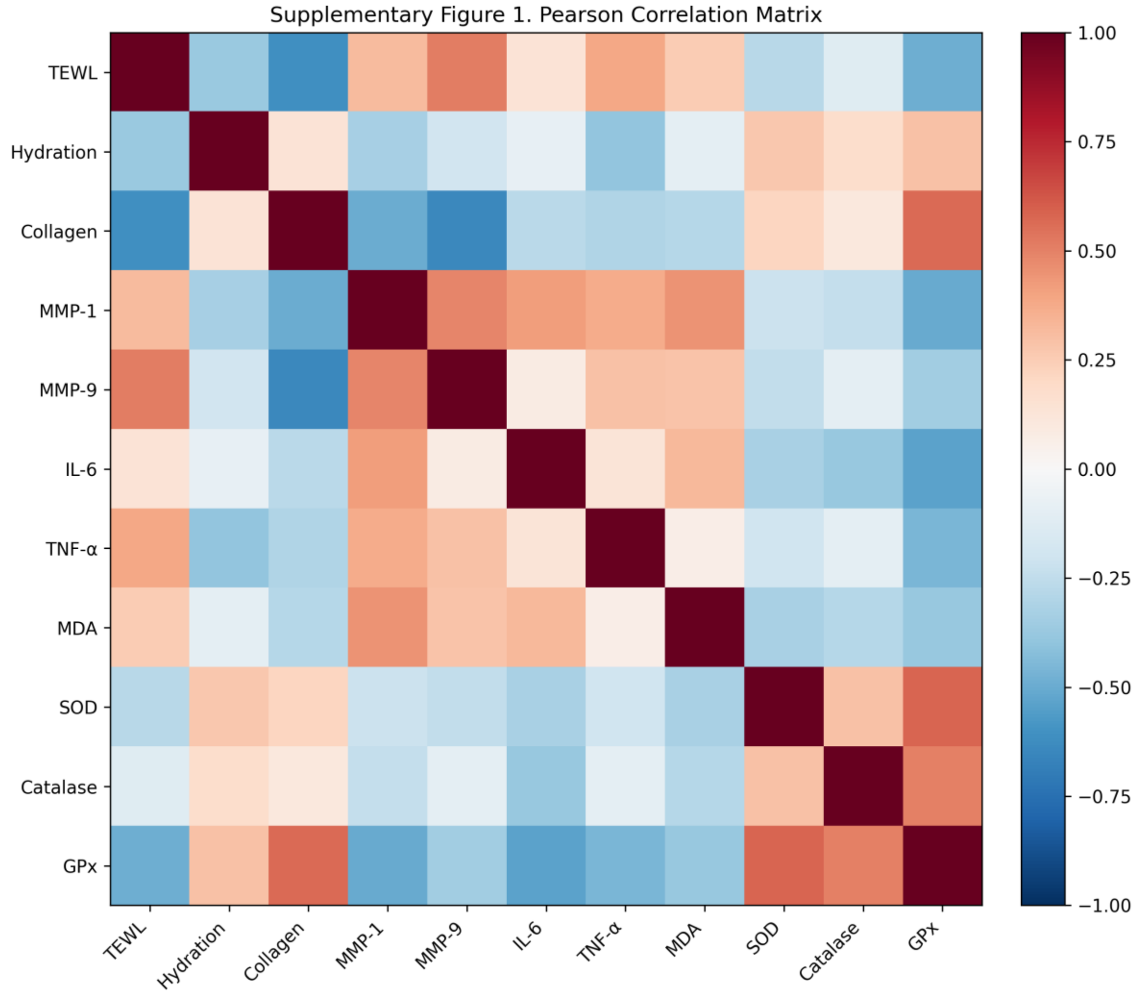

Numerical values (mean ± SEM) of skin biophysical parameters, barrier components, extracellular matrix–related markers, inflammatory cytokines, and oxidative stress indicators in UVB-irradiated hairless mice treated with standardized *Centella asiatica* extract.
